# Hypertension: From Epidemiology to Therapeutics

**DOI:** 10.1155/2012/908431

**Published:** 2012-03-25

**Authors:** Manuel Velasco, Zafar Israili

**Affiliations:** ^1^Vargas Medical School, Clinical Pharmacology unit, Universidad Central de Venezuela, Carcas 1041, Venezuela; ^2^Department of Medicine, Emory University School of Medicine, Atlanta, GA 30322, USA

P. Boli and R. Campbell discuss whether recommendations for the management (and control) of high blood pressure really result in improved cardiovascular (CV) outcomes. Adherence to the recommendations by the Canadian Hypertension Education Program did significantly improve all the aspects (awareness, treatment, and control) of hypertension, as well as mortality and hospitalization due to strokes, heart failure, and myocardial infarction. The authors suggest that application of similar recommendations for the management and control of hypertension in other regions of the world may be helpful. An interesting paper by C. Sierra reviewed that presence of cerebral white matter lesions (usually found in the aged population) were more severe and appeared early in patients with high blood pressure. There was an association between blood pressure parameters obtained by ambulatory blood pressure monitoring (ABPM) and presence of white matter lesions, suggesting that data obtained from ABPM may be helpful in identifying asymptomatic hypertensive patients with brain damage. R. Fagugli and C. Taglioni discuss that aldosteronism (hyperaldosteronism), which was thought to be rare (affecting 1% of the hypertensive population), occurs at a much higher incidence (5–20%) in patients with type 2 diabetics and resistant hypertension and is associated with a high incidence of cardiovascular (CV), cerebrovascular, and kidney complications. The variation in the estimates of prevalence may be due to methodology and definitions. M. Roy et al. determined that among African Americans with type 1 diabetes mellitus, without elevated blood pressure, 29% develop hypertension in the course of 6 years. The risk factors associated with the development of hypertension include age, duration of diabetes, family history of hypertension, higher baseline arterial pressure, overt proteinuria, presence of retinopathy or peripheral neuropathy, smoking, and psychological factors, such as perceived hostility. An interesting paper by M. Yamori et al. investigated the association of hypertension with periodontitis and/or tooth loss (apparently from periodontal disease) in a group of nonsmoking, nondrinking middle-aged African women. Their studies show that the severity of periodontal disease was significantly associated with increased systolic and diastolic blood pressure. In addition, low dietary intake of potassium and fiber was directly associated with higher blood pressure and periodontal inflammation. On the other hand, higher dietary intake of potassium, fruits, and vegetables decreased inflammation of the gums. The authors suggest that increased dietary intake of potassium, fruits, and vegetables may decrease the incidence of periodontitis. Q. Nguyen and coworkers analyzed cardiovascular risk factors in a large cross-sectional survey in Vietnam. The prevalence of the clustering of metabolic (such as in the metabolic syndrome) and behavioral risk factors was more prevalent in men than in women, and there was a 4-fold higher overall 10-year CV risk in men than in women. Targeting only a single risk factor would not decrease the overall risk. A pharmacological means of correcting endothelial dysfunction was proposed by M. Pokrovskiy et al., by the use of inhibitors of arginase (which catalyses the degradation of L-arginine and thereby reducing the production of nitric oxide), thus increasing the production of the vasodilating nitric oxide, thereby protecting the endothelium. In their studies in rats, R. Peroni and coworkers observed that phytoestrogens produced an estrogen receptor-dependent enhancement of anandamide-induced reduction of contractility (of mesenteric bed) caused by noradrenaline by modulation of calcitonin gene-related peptide. The in vivo suppression of adrenergic hyperactivity (which precedes the onset of hypertension) by anandamide (such as by oral administration of genistein or daidzein) was observed in female but not in male rats or ovariectomized female rats.

*Manuel Velsco* *Manuel Velsco* *Zafar Israili* *Zafar Israili*

